# Dynamic 3D metasurface holography via cascaded polymer dispersed liquid crystal

**DOI:** 10.1038/s41378-024-00855-6

**Published:** 2024-12-24

**Authors:** Shuo Sun, Jin Li, Xiaoxun Li, Xianyu Zhao, Kun Li, Liang Chen

**Affiliations:** 1 College of Optical and Electronic Technology, China Jiliang University, Hangzhou, 310018 China; 2 School of Instrumentation and Optoelectronic Engineering, Beihang University, Beijing, 100191 China; 3 Science and Technology Center for Quantum Biology, National Institute of Extremely-Weak Magnetic Field Infrastructure, Hangzhou, 310051 China; 4 Beihang Hangzhou Innovation Institute, Hangzhou, 310052 China; 5 Joint International Research Laboratory of Information Display and Visualization, School of Electronic Science and Engineering, Southeast University, Nanjing, 210000 China; 6 CamOptics (Suzhou) Ltd., Suzhou, 215000 China

**Keywords:** Micro-optics, Electrical and electronic engineering

## Abstract

Metasurface with natural static structure limits the development of dynamic metasurface holographic display with rapid response and broadband. Currently, liquid crystal (LC) was integrated onto the metasurface to convert the passive metasuface into an active one. But, majority of LC-assisted active metasurfaces often exhibit trade-offs among degree of freedom (DoF, typically less than 2), information capacity, response speed, and crosstalk. Herein, at first time, we experimentally demonstrate a cascaded device with polymer dispersed liquid crystal (PDLC) and broadband metasurface, enabling dynamic three-dimensional (3D) holographic display with ultra-high contrast, rapid response and continuous regulation. The PDLC droplets enable modulation of scattering state of incident light by high-speed dynamic control system for electric scanning. Based on self-addressing, rapid response and multi-channel PDLC-metasurface device, the dynamic holographic effect of monochrome holographic images switching and color-changing holographic display with broadband, low-crosstalk and high contrast, has been achieved. Our approach offers a novel perspective on dynamic metasurface.

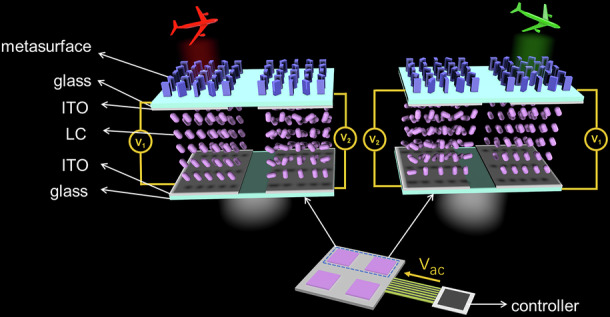

## Introduction

Metasurfaces, with their two-dimensional (2D) sub-wavelength meta-atomic structure for local phase and amplitude modulation of the light field, enable effective detection and interaction with incident light within a limited size range, providing micro- and nano-pixel modulation of the light field^1–3^. This presents a significant potential for advancing optical components with trade-offs between size, weight, and power consumption, providing a novel solution for compact flat optical device design^4–7^. The ultra-small size metasurface offers integration capabilities with other devices compared to conventional optical devices, enabling the achievement of miniaturized high-performance devices with multi-functions, such as wavefront engineering^8–10^, polarization transformation^11–13^, generation of optical vortices^14–16^, and three-dimensional (3D) holography^17,18^. Especially, as a medium of hologram, metasurface can satisfy the record and reconstruction of more details in 3D visual cues, making it the most suitable technology currently available for achieving photorealistic 3D holographic displays. However, to date, the majority of reported metasurface holograms only exhibited static characteristics and featuring fixed optical responses^19–21^, which limit the development of dynamic 3D holographic display. In order to fulfill the requirements of intelligent and adaptive display systems, it is crucial to develop newly active metasurface that enable real-time dynamic modulation of the light field.

Currently, three kinds of active metasurface can be implemented into dynamic 3D holography: multiplexing metasurface, structural modification metasurface and integrated metasurface. The first active metasurface encompasses a multiplexing function to effectively respond to variations in the fundamental parameters of incident light fields, including the incident light’s wavelength^22^, polarization^23,24^, orbital angular momentum^25^, and other properties. The design of metasurfaces corresponds to the changes in the incident light field, allowing for dynamic 3D holography by altering the incident light fields. The integration of multiple states of incident light, such as wavelength and polarization^26^, or wavelength and orbital angular momentum^27^, enables a broader range of dynamic states to be achieved. The second involves some passive modulation techniques that induce structural modifications within the metasurface through chemical reaction or mechanical deformation^28–30^. The second type typically employs materials with distinct characteristics, such as vanadium dioxide (VO_2_)^31^, magnesium (Mg)^32^, polydimethylsiloxane (PDMS)^33^, and hydrogel^34^, to fabricate the active metasurface. Via external stimuli (environmental changes), the size, period, or material refractive index of the metasurface unit structure can be dynamically adjusted to regulate the phase generated, thereby achieving variations in holographic images. However, its intricate processing and limited response speed in control hinder its practical applicability. Lastly, the third active metasurface involves the utilization of active modulation techniques, which allow for precise and dynamic manipulation of incident light parameters through the integration of components such as liquid crystal (LC), microfluidics, and digital electronic control^35–37^. It can also be combined with multiplexing techniques, such as utilizing electrically controlled LC to manipulate the polarization^38^ or wavelength^39^ of incident light, in conjunction with the corresponding responsive metasurface. Owing to its convenient control and rapid response speed, this active metasurface has emerged as a prominent research area for achieving dynamic 3D holography. However, further investigations are warranted to broaden the range of integrated devices and accomplish multifunctional integration in dynamic holographic displays^40,41^.

As a prototypical component which can integrated with metasurface holography, LC exhibits high-effective modulation capability of light field and simple actuate process, however, most of LC devices only have polarization-converted ability which suffering from limited number of channels, severe crosstalk and subpar imaging quality for 3D holography^42–44^. The polymer dispersed liquid crystal (PDLC) is a novel class of LC that finds applications in optical switches, light waveguides, and dynamic windows^45,46^. It comprises low molecular weight LC and high molecular weight polymers in a phase-separated state and the LC droplets are uniaxial spherical crystals with a bipolar molecular orientation distribution. In the absence of an external electric field in the PDLC, the alignment of LC droplets is randomly dispersed, leading to a refractive index mismatch between the effective refractive index and that of the polymer matrix. When incident light enters PDLC without stimulation, multiple reflections and refractions occur at the interface between LC droplets and the polymer matrix, resulting light in PDLC exhibiting a scattering state; While, as a sufficiently strong electric field intensity applied to the PDLC, the LC droplets align themselves along the direction of the electric field, resulting in a refractive index matching that of the polymer substrate. Consequently, the incident light can smoothly and totally transmit through PDLC, becoming rendered transparent to it^47–49^.

**Fig. 1:**
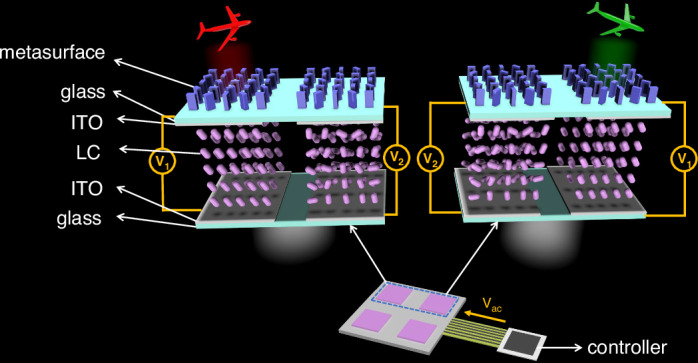
Schematic illustration of the electrically tunable PDLC-metasurface dynamic 3D holographic display

In this work, at first time, we experimentally demonstrate a cascaded device with PDLC and broadband metasurface, enabling dynamic 3D holographic display with ultra-high contrast, rapid response and continuous regulation under entire visible spectrum. As depicted in Fig. 1, the design is based on the integration of vertically stacked space channel metasurface and PDLC in a cascaded manner, in which the PDLC droplets enables modulation of the scattering state (reflections and refractions) of incident light by a high-speed dynamic control system for electric scanning. Based on it, a self-addressing, rapid response and multi-channel (four) PDLC-metasurface device was fabricated to display 3D holography with broadband, low-crosstalk and high-contrast. Besides, the four spatial channels are composed of optimized a-Si nanocolumns, achieving modulation efficiency that is more than 40% under entire visible spectrum. By precisely controlling the thickness of pixelated PDLC and aligning it with the metasurface, each pixel can be independently controlled through individual indium tin oxide (ITO) electrodes, thereby maintaining a minimum response time of 19.97 ms and achieving minimal crosstalk between pixel channels. As a result, the dynamic holographic effect of monochrome holographic images switching and color-changing holographic display has been achieved. Our approach offers a novel perspective on dynamic metasurface, which can be extensively applied in the fields of optical communication, optical encryption, optical storage, and laser manufacturing.

## Results

### The preparation of PDLC device with high contrast and rapid respond

**Fig. 2:**
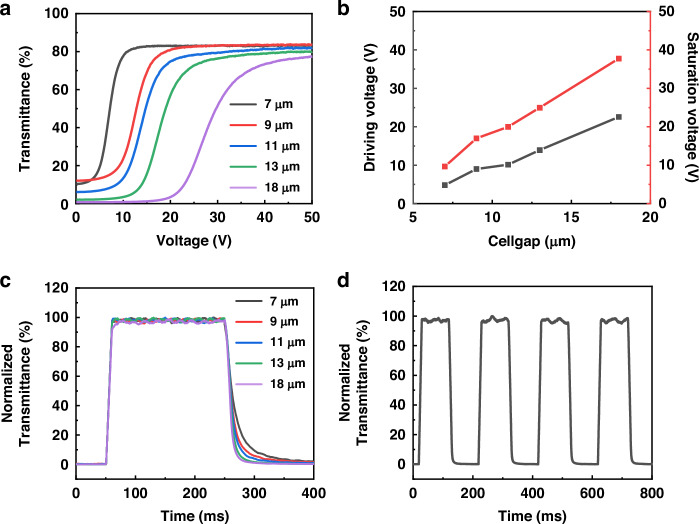
Applied voltage-transmittance performance characterization of PDLC devices with increased thicknesses (from 7 μm to 18 μm) under wavelength of 635 nm laser source. **a** The voltage-dependent transmittance curves between different thickness of PDLC devices. **b** Drive voltage and saturation voltage as a function of different thickness of PDLC devices, showing that both of them increased with the thickness raising. **c** The switch on and off rate of PDLC devices with various thickness. **d** The circle of switch on and off process of PDLC device with the thickness of 13 μm

The PDLC devices were fabricated by injecting dispersed LC droplets into a polymer matrix wherein polyacrylate polymers are blended with LC to achieve homogeneous solution and the solution was subsequently sandwiched between two layers of ITO substrate (details in Materials and methods). In the PDLC encapsulation process, the thickness (cell gap between two ITO substrates) of the PDLC devices were precisely regulated within a range of 7 µm to 18 µm by incorporating spherical spacer particles with varying sizes. At first, the performance characterization of PDLC devices with different thicknesses and various applied voltage under a wavelength of 635 nm laser source were presented. As shown in Fig. 2a, the transmittance of all PDLC devices was minimal, rendering them opaque, when no electric field stimulation was applied. This can be attributed to the random orientation of LC molecules, resulting in light scattering when passing through the film (Fig. S1a). Once applied voltage to this PDLC device, the LC molecules in it were reduced into specific alignment (Fig. S1b), leading to progressive increase of transmittance until it reaches the maximum value. Therefore, the thickness of PDLC device is a crucial factor that significantly influences both the driving voltage (defined as the voltage at which PDLC achieves its maximum transmittance of 10%) and saturation voltage (defined as the voltage at which PDLC reaches its maximum transmittance of 90%).

For different thickness of PDLC devices, the overall trend of the transmittance remains consistent by increasing applied voltage, however, discernible differences exist in the minimum and maximum transmittance of them, such as the minimum and maximum of 7 μm thickness is 10.3% and 83.5%; 9 μm is 12.1% and 83.4%; 11 μm is 6.1% and 82.3%; 13 μm is 2.1% and 80.4%; 18 μm is 0.7% and 79.0%, respectively. The statistics of drive voltage and saturation voltage as function of thickness of different PDLC devices with increased thickness (from 7 μm to 18 μm) were presented in Fig. 2b, which is showing that both of them increased with the thickness raising. This is because the process of driving PDLC devices requires applying a sufficient electric field to rearrange the LC molecules. As the thickness of PDLC increases, higher voltage is needed to maintain sufficient electric field strength inside the material. In addition, as the thickness of PDLC devices increases, the distribution range of LC molecules expands. The process of rearranging molecules requires a stronger electric field to cover a larger response area. The switch-off state is compromised when the thickness is too low (<11 μm) due to its subpar scattering effects; whereas a higher thickness (>13 μm) not only diminishes light transmittance in the switch-on state but also amplifies adjustment complexity (higher drive voltage and saturation voltage). The transmittance, drive voltage and saturation voltage variation curves of PDLC under other laser wavelength of 473 nm and 532 nm are basically identical (Fig. S2 and Fig. S3). To achieve precisely dynamic control, it is imperative to ensure a high contrast (ratio of maximum transmittance to minimum transmittance of PDLC device), while reducing the driving voltage and saturation voltage can facilitate the operation of PDLC devices. Therefore, through all experimental data, it has been determined that the optimal thickness parameter is ca. 13 μm, which exhibited a minimum transmittance below 5% and clearly indicating the off-state, while necessitating lower applied voltage for achieving maximum transmittance. Additionally, the response speed can be analyzed by examining the normalized transmittance (defined as the ratio of the difference between the transmittance and the minimum transmittance to the difference between the maximum and minimum transmittance) change curve (Fig. 2c). The results indicate that the rise time of PDLC with different thicknesses remains consistently below 10 ms, thereby demonstrating its rapid switching speed. The switching speed, however, exhibits a deceleration as the thickness of PDLC decreases, yet fall time consistently remains below 50 ms (details in Supporting Information Note 4 and Fig. S4). When the thickness of PDLC decreases, it increases the constraint force on LC molecules in the matrix, resulting in a weakened restoring force after power-off. This leads to a longer time for LC molecules to recover from an ordered arrangement to a disordered state, thus slowing down the descent speed. The multiple repeated switching process of the 13-um PDLC demonstrates consistent response speed and maintains maximum/minimum transmittance, as depicted in Fig. 2d, showcasing exceptional dynamic control performance. Therefore, by adjusting the thickness of PDLC devices, we can obtain transmittance control devices with high switching ratio and rapid response ability.

**Fig. 3:**
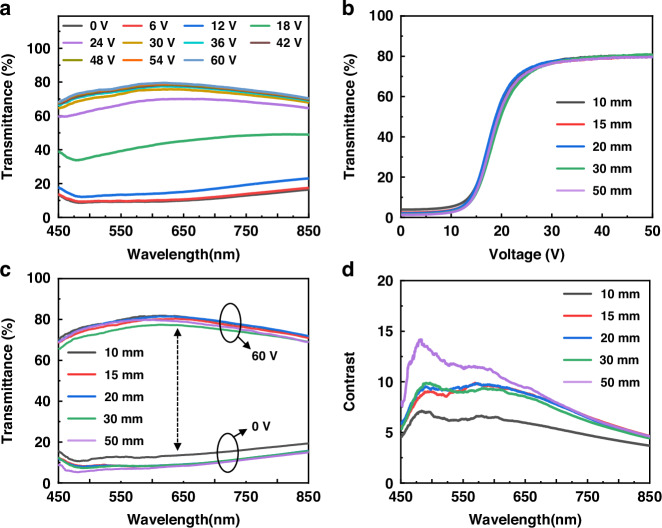
The test distance and broadband evaluation of PDLC devices indicting high switch ratio performance. **a** With the increase of the applied voltage, the transmittance change diagram of PDLC device (thickness = 13 μm) in visible wavelength range shows that when voltage < 12 V, the transmittance is lower than 20%, while voltage > 24 V, it has a transmittance higher than 60%. **b** The voltage-dependent transmittance of PDLC of variations at different test distances between detector and PDLC. **c** Transmittance of PDLC under different test distances (10 mm ~ 50 mm) at various wavelengths (450 nm ~ 850 nm) without voltage application (0 V) and with saturated voltage (60 V), showing high contrast across entire visible spectrum. **d** Contrast of PDLC device under different test distances at various wavelengths without voltage application and with saturated voltage

The contrast is a crucial performance parameter for LC devices. So, in this study, at optimal thickness (13 μm), with the increase of the applied voltage, the transmittance change diagram of the PDLC device, in visible wavelength range, was characterized, which showed that when voltage <12 V, the transmittance is lower than 20%; while voltage >24 V, it has a transmittance higher than 60% (Fig. 3a). In the entire visible spectrum, the transmittance of PDLC remains minimal and unchanged before reaching the driving voltage, irrespective of voltage variations. Subsequently, upon reaching the driving voltage, a gradual increase in PDLC’s transmittance was observed across different wavelengths. Once saturation voltage was attained, no further changes in transmittance. Besides, the variations in transmittance of PDLC devices with other thicknesses under various wavelengths and voltages are illustrated in Fig. S5, which were all conformed to the above rule. When the voltage changes, the PDLC basically does not change the polarization of the incident light (Fig. S6). In PDLC devices, LC molecules are randomly distributed in a scattering state that does not selectively affect the polarization of light. When voltage is applied, the arrangement of LC molecules becomes ordered, enabling light to pass through. However, this alignment of LC molecules does not introduce any specific polarization conversion effect. Therefore, the PDLC device can achieve high contrast via a simple voltage change.

Additionally, due to the scatting light ability of PDLC, the gap between integrated PDLC-metasurface also affects contrast of the output image and flexible dynamic regulation. Here, the transmittance as a function of voltage with different test distance (the distance between the PDLC device and the detection point) was also conducted to find the optimal integrated gap. As depicted in Fig. 3b, under a 635 nm laser, the transmittance of PDLC exhibits uniformity across various test distances ranging from 10 mm to 50 mm, demonstrating a consistent trend. A similar observation is made for incident lasers with wavelengths of 473 nm and 532 nm respectively (Fig. S7). Moreover, in Fig. 3c, the transmittance of PDLC under different test distances (10 mm ~ 50 mm) at various wavelengths (450 nm ~ 850 nm) without voltage application (0 V) and with saturated voltage (60 V), showing high contrast across entire visible spectrum. The contrast was summarized in Fig. 3d, which is showing that the decreased in test distance may lead to a marginal decline in contrast. With a decrease in test distance, the scattering path of light becomes shorter, resulting in a small portion of scattered light entering the detector. Additionally, the shorter test distance limits the uniformity of beam diffusion during transmission, affecting the consistency of light intensity in transparent states. However, the overall level remains significantly high (more over than 5), leading the PDLC are suitable for integration with metasurface. Therefore, the integration of PDLC with metasurface effectively enable the broad spectral and rapid response, high contrast and continuous control of light field to achieve active dynamic metasurface.

### Optimization of meta-atoms for broadband and high-efficiency metasurfaces

**Fig. 4:**
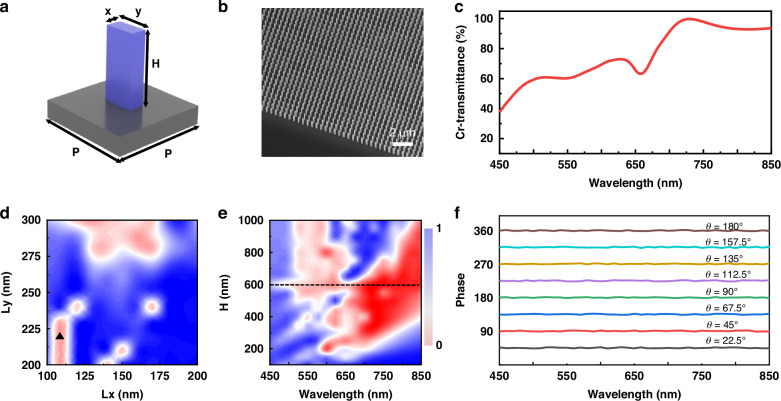
The optimization of nanopillar parameters for achieving high-efficiency broadband metasurface. **a** The 3D illustration of a single unit cell (P) exhibiting a rectangular nanopillar with dimensions of width (x), length (y), and height (H) coated on a transparent thick SiO_2_ film. **b** Aerial-view SEM image of the broadband metasurface. **c** Cross-polarization transmittance (cr-transmittance) of the α-Si based nanopillar structure with optimized parameters. **d** Simulation graph of the cr-transmittance of a rectangular nanopillar with different length-width ratio at the optical wavelength of 635 nm and **e** of height changing of nanopillar at different wavelengths from 450 nm to 850 nm. **f** Phase generated by nanopillar at different angles under wavelengths of 450-850 nm

The metasurface that responds to the entire visible range has a wider range of applications when integrated with the broadband PDLC devices, therefore, the corresponding broadband metasurface was designed, simulated and characterized with its optical properties. The metasurface utilizes amorphous silicon (α-Si) as the unit structural material, exhibiting high efficiency within the visible light spectrum. The efficiency is the cross-polarization transmittance (cr-transmittance) of the metasurface, defined as ratio of the intensity of light with the opposite chirality of the incident light to the intensity of the incident light. The metasurface was fabricated on the top layer of PDLC glass using precise photolithography positioning and etching technology to create adjacent nanopillar with a period of P = 500 nm, in which the unit structure as depicted in Fig. 4a, exhibits a nanopillar characterized by dimensions of x (width), y (length), and H (height). Figure 4b is the scanning electron microscope (SEM) image with aerial-view showing the nanopillar arranged in a periodic pattern in the metasurface. By optimizing the nanopillar dimensions while maintaining the simplicity of unit structures, it is possible to enhance cr-transmittance. The optimized simulation values for cr-transmittance are depicted in Fig. 4c, demonstrating sustained high cr-transmittance exceeding 40% within the entire visible spectrum. The optimization of the metasurface was achieved through the size optimization of a single nanopillar utilizing the finite-difference time-domain method (FDTD). The cr-transmittance of the nanopillar was simulated by varying the lengths of x and y at a wavelength of 635 nm, as illustrated in Fig. 4d. Besides, the cr-transmittance at different nanopillar sizes at 473 nm and 532 nm, is depicted in Fig. S8. When Lx = 110 nm and Ly = 220 nm, it was observed that the cr-transmittance reached its maximum value, surpassing 50% in all simulated results. The cr-transmittance of nanopillar height across the entire visible spectrum was characterized, based on which it was determined that H = 600 nm exhibited optimal performance (Fig. 4e). The metasurface employs Pancharatnam-Berry, of which the assign a rotation angle θ to each nanopillar at intervals of 22.5°, resulting in a phase of 2θ. By rotating the nanopillar from 0-180°, the metasurface covers a complete phase range of 0-360°. As depicted in Fig. 4f, it is observed that the nanopillar of identical angle exhibit consistent phase under the entire visible spectrum, ensuring a homogeneous holographic image quality across a broad range of wavelengths. Therefore, the prepared metasurface with the performance of broadband response and high efficiency was enabled, and it can be well applied to integrated with PDLC devices and generate the dynamic 3D holography.

### Continuously modulated holographic display based on PDLC-metasurface

**Fig. 5:**
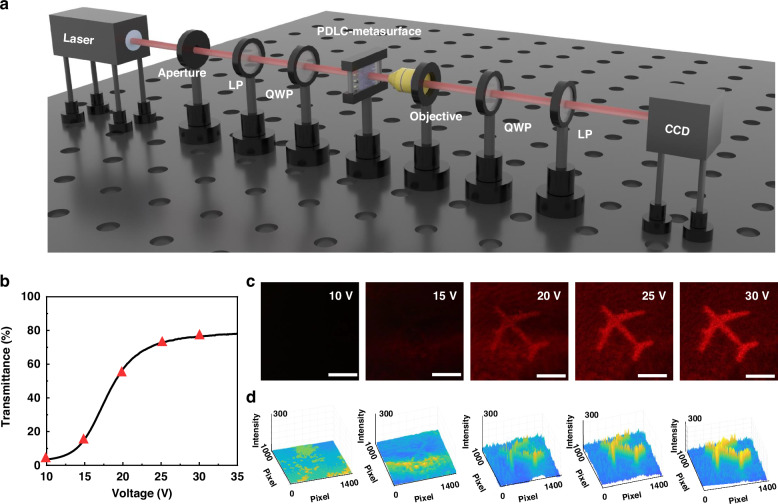
The reconstruction of dynamic 3D holography with light intensity modulation via PDLC-metasurface. **a** Schematic illustration of the experimental setup for characterizing the light intensity regulation of 3D holography, which comprised a laser source, aperture, linear polarizers (LP), quarter-wave plate (QWP), PDLC-metasurface, objective lens, and camera (CCD). **b** PDLC device exhibits continuous transmittance modulation in response to voltage (10 V ~ 30 V), enabling dynamic light intensity adjustment of 3D holography **c** CCD captured image of 3D holography (the scale bar represents 2 cm) with different light intensity in a real time **d** and its corresponding simulated values

The integrated PDLC-metasurface (the processing details in Materials and methods and Fig. S9) has the ability to reconstruct dynamic 3D holography with light-intensity modulation. We developed a holographic reconstruction system for PDLC-metasurface couplers, which utilizes lasers, an aperture, linear polarizers, quarter-wave plates, and an objective lens to establish the optical path system. And the PDLC-metasurface was driven by a signal generator and power amplifier, enabling the reception of holographic images through CCD, as depicted in Fig. 5a. We utilize a 3D metasurface holographic display technology based on holographic lens. The algorithm for computer-generated holograms utilizes holographic lens to incorporate phase cues from 3D images at various depths, along with virtual holographic-lens featuring multiple layers of varying focal lengths. The airplane is categorized into three layers according to depth, and the CCD is maneuvered forward and backward for observation. Different depth and color holographic images of airplanes are displayed in Fig. S10. The number of layers can be adjusted according to requirements. In order to facilitate CCD observation and effect display, subsequent studies loaded the airplane image to the same depth. The experiment exhibits an efficiency (defined as the ratio between the total optical power of the zero-order filtered holographic image and the optical power of the incident light) of 66.68% at 635 nm, 57.49% at 532 nm, and 48.48% at 473 nm (details in Supporting Information Note 11). The experimental efficiency exceeds 90% of the simulated efficiency, with a slight reduction possibly attributed to losses in optical components or imperfections in metasurface fabrication.

Because of the integrated PDLC device, the passive metasurface was transformed into an active controllable one, enhancing the versatility dynamic 3D holographic manipulation method. Here, the transmittance as the function of voltage was characterized to prove that the PDLC device was approximatively continuous modulated by adjusting the voltage (Fig. 5b), resulting in gradual change in the intensity of the holographic image depicting an airplane, as illustrated in Fig. 5c. And the holographic phase map and its simulated reconstruction image of metasurface were supported in Fig. S11. As the voltage increases from 10 V to 30 V, gradual enhancement in the intensity of the holographic image of airplane was observed, exhibiting remarkable contrast between the off state at 10 V and the on state at 30 V. By fourier transform calculation and simulation of light intensity of holographic images, the light intensity of them were further elucidated. The 3-D surface plots light intensity for the same holographic image at different external electric field indict the corresponding intensity distributions intensify with increasing voltage (Fig. 5d), which is corresponding to the switch-on process with ultra-high contrast. These experiments proved that the PDLC-metasurface can rapidly and continuously control the light intensity of holographic image for dynamic 3D holography. Especially, the integration of PDLC-metasurfaces devices which exhibit continuous controllable adjustment and high contrast, differs from traditional polarization LC devices that exhibit only two states and possess low contrast between these states leading to significant crosstalk. Thereby, the prepared PDLC-metasurface has the ability to achieve a high-quality 3D holographic display.

### The broadband and low-crosstalk multichannel dynamic PDLC-metasurface holographic display

**Fig. 6:**
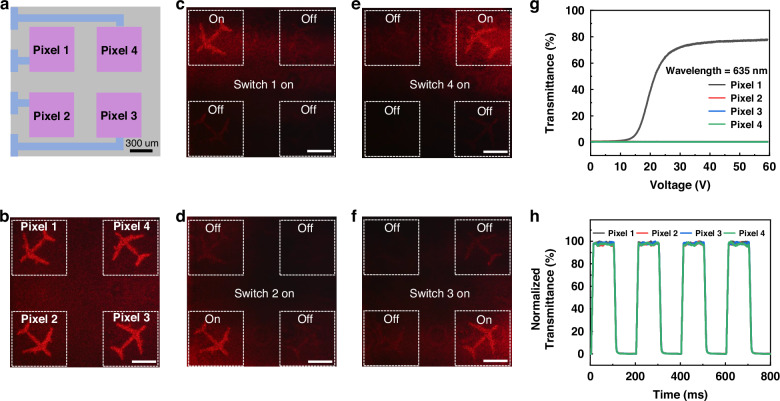
The preparation of multiple channel dynamic addressing 3D holographic display. **a** Schematic cartoon illustrating the four-pixel channels (each pixel channel with the size of 500 μm) of PDLC device and operation of each pixel is controlled by microcircuits (blue). **b** The operation of the four channels dynamic addressing 3D holography display with four flying airplane meaning four pixel channels were switch on state (the scale bar represents 0.5 cm). **c–f** 3D holographic image sequential switchover exhibition (from pixel 1 to pixel 4) of the four-channel 3D hologaphic display proving its dynamic addressing performance (the scale bar represents 0.5 cm). **g** When Pixel 1 was activated, the transmittance of each pixel was compared under the wavelength of 635 nm, which is indicating that the crosstalk between 1 and other 2, 3, 4 channel was near in zero. **h** The transmittance response through the four-pixel channels were cyclically modulated, resulting in periodic switch on and off states

Based on the mechanism of PDLC-metasurface device, a multiple-channel dynamic addressing 3D holographic display was prepared with high-performance via metasurface cascaded with corresponding pixelated PDLC. The metasurface design was spatially multiplexed and corresponded to the four-channel pixels. This holographic display, as illustrated in Fig. 6a, was designed with four channels, each of which were independently controlled based on pixelated ITO electrodes, where the four-pixel channels (each pixel channel with the size of 500 μm) of PDLC device (purple) and operation of each pixel is controlled by microcircuits (blue). As depicted in Fig. 6b, the dynamic 3D holographic display of four channels was reconstructed using a 635 nm laser, where a holographic image of airplanes in different orientations was performing a loop maneuver. This presentation offers enhanced spatial information and an expanded FoV. Because of the four independent control of each spatial channel, this dynamic display, as illustrated in Fig. 6c–f, allows for the switching display of different holographic images and facilitates dynamic addressing with high contrast. The PDLC-metasurface demonstrates a broadband response ability, as illustrated in Fig. S12, exhibiting holographic images and dynamic control responses upon illumination by lasers at 473 nm and 532 nm, respectively. The results depicted in Fig. 6g reveal that the activation of a single channel leads to an impressive transmittance exceeding 80%, while the remaining pixels exhibited transmittance levels below 0.06%. The transmittance of the four channels, which are sequentially activated at different wavelengths, is depicted in Fig. S13 and Supporting Information Note 14. Through further analysis of the total optical power of zero-order filtered holographic images and the optical power of other channels, we can evaluate the low crosstalk exhibited by holographic displays. The experiment demonstrates that the total optical power of holographic images exceeds 22 times that of the deactivated channel’s optical power, resulting in a significant enhancement in contrast. In addition, based on the average pixel intensity of the holographic images (Fig. 6c–f and Fig. S12c, d), the crosstalk between channels can also be analyzed. The average pixel intensity of activated holographic image is more than 13 times that of deactivated channels. When a channel is activated, the transmittance of adjacent channels is extremely low (details in Supporting Information Note 15). The channels exhibit low crosstalk among one another. The normalized transmittance, as depicted in Fig. 6h, demonstrates consistent maximum and minimum transmittance levels for each pixel channel, along with uniform response speed (details in Supporting Information Note 16). This characteristic facilitates rapid switching display of holographic images with homogeneous intensity. The response of each pixel of the PDLC-metasurface under illumination by lasers with wavelengths of 473 nm and 532 nm is depicted in Fig. S14.

**Fig. 7:**
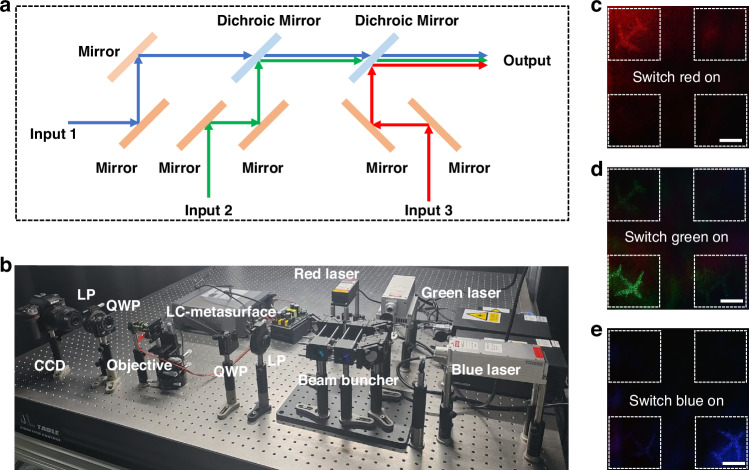
The preparation of broadband dynamic 3D PDLC-metasurface holographic display. **a** The optical path schematic is constructed by combining a 473 nm laser (input 1), a 532 nm laser (input 2), and a 635 nm laser (input 3) through the utilization of reflector mirrors and dichroic mirrors. **b** Image of the integration of multiple colored light beams in a holographic reconstruction optical path. **c–e** A demonstration of dynamically broadband-addressed 3D holographic display (the scale bar represents 0.5 cm)

Due to the broadband respond and spatial multiplexing ability of the PDLC-metasurface device, high-performance broadband 3D dynamic holographic display was experimentally conducted here under combining the 473 nm, 532 nm, and 635 nm lasers through the utilization of mirrors and dichroic mirrors (Fig. 7a). Aligning the beam with the multi-channel PDLC-metasurface pixels, as depicted in Figs. 7a and 7b, encompassing both a schematic diagram and an image of the optical path. By implementing voltage-driven control on individual pixels, holographic image airplane achieved dynamic addressing by switching the display between multiple pixels. The holographic image demonstrates the transition from red to green, ultimately transforming into blue while maintaining consistent image quality, as illustrated in Fig. 7c–e. Compared with other research on electrically controlled dynamic metasurface holography, our work has the advantages of fast response, high contrast, and continuous modulation (details in Supporting Information Note 17). This method provides a new idea for preparing broadband dynamic 3D holography.

## Discussion

In conclusion, we experimentally demonstrate a cascaded device comprising of PDLC and broadband metasurface. This integration enables the realization of dynamic 3D holographic display with ultra-high contrast, rapid response, and continuous regulation across the entire visible spectrum. The design is based on vertically stacked space channel metasurfaces and PDLC in a cascaded manner. The PDLC droplets allow for modulation of the scattering state (reflections and refractions) of incident light through a high-speed dynamic control system for electric scanning. Based on this concept, we have fabricated a self-addressing, rapid response, multi-channel (four) PDLC-metasurface device capable of displaying broadband 3D holography with low crosstalk and high contrast. As a result, we have achieved dynamic holographic effects such as monochrome holographic images switching and color-changing holographic displays. Our approach offers a novel perspective on dynamic 3D holographic display.

## Materials and methods

### PDLC preparation

PDLC was fabricated through the phase separation by polymerization, wherein polyacrylate polymers were blended with LCs to achieve a homogeneous solution. The LC mixture demonstrated a nematic LC phase at ambient temperature. Through polycondensation, the molecular weight increased until it reached a critical size at which their mutual solubility decreases, resulting in phase separation. LCs form droplets and gradually grow, ultimately becoming immobilized through polymerization into solidified polymers. By modulating the intensity of light curing, precise control over the reaction rate can be achieved, thereby exerting influence on the size and morphology of droplets. The two-layer pixelated ITO was sequentially cleansed using deionized water, acetone, and alcohol before being encapsulated to form a LC enclosure. Interval particles of varying diameters were used between the two ITO glass substrates to fabricate the LC cell. The purpose behind interval particles was to regulate the thickness of the LC and ensure thickness uniformity. The PDLC solution mentioned above was carefully introduced into the LC cell, subjected to ultraviolet irradiation, and subsequently, the filling port was thoroughly cleansed. This procedure results in the formation of PDLC.

### PDLC-metasurface devices preparation

The metasurface was prepared on the glass of ITO on the top layer of PDLC. The α-Si nanorod was utilized to fabricate the dielectric metasurfaces on substrate. To begin with, a layer of α-Si film with a thickness of 600 nm was fabricated using plasma-enhanced chemical vapor deposition. The resist layer of poly-methyl-methacrylate (PMMA) is applied through spin-coating onto the α-Si layer. Reheat at 180°C for two minutes in order to eliminate any residual solvents. Employ electron beam lithography (EBL) technology for the fabrication of desired metasurface patterns. Draw a grid based on the known ITO pattern and achieve alignment of metasurface and pixel electrode using SEM in the EBL system. With an alignment accuracy of 100 nm, it meets the requirements for PDLC control of metasurfaces. Develop the pattern in a mixed solution of MIBK and IPA with a ratio of 1:3, then clean with IPA. Afterward, a layer of chromium with a thickness of 100 nm was deposited using electron beam evaporation. Following this, the excess layers were eliminated by immersing them in heated acetone during the lift-off procedure. Finally, the intended configuration was transferred from chromium to α-Si through the utilization of inductively coupled plasma reactive ion etching. The entire of process and alignment does not require additional equipment, which does not significantly increase the difficulty of preparation, promoting large-area production and commercial applications.

## Supplementary information

Supporting information
