# Supplementary material for: Dynamic 3D metasurface holography via cascaded polymer dispersed liquid crystal
Source: Microsyst Nanoeng. 2024 Dec 24;10:203. doi: 10.1038/s41378-024-00855-6 (PMC11668883; doi:10.1038/s41378-024-00855-6)
Supplement: Supplementary file 1 — Supporting information [file 41378_2024_855_MOESM1_ESM.docx]

Supporting Information

Dynamic 3D metasurface holography via cascaded polymer dispersed liquid crystal

*Shuo Sun,* *^1, 2, 3, 4^ Jin Li, ^2, 3, 4, *^ Xiaoxun Li, ^3, *^* *Xianyu Zhao, ^5^ Kun Li, ^6, *^ Liang Chen ^1^*

^1^College of Optical and Electronic Technology, China Jiliang University, Hangzhou 310018, China

^2^School of Instrumentation and Optoelectronic Engineering, Beihang University, Beijing 100191, China

^3^Science and Technology Center for Quantum Biology, National Institute of Extremely-Weak Magnetic Field Infrastructure, Hangzhou 310051, China

^4^Beihang Hangzhou Innovation Institute, Hangzhou 310052, China

^5^Joint International Research Laboratory of Information Display and Visualization, School of Electronic Science and Engineering, Southeast University, Nanjing 210000, China

^6^CamOptics (Suzhou) Ltd., Suzhou 215000, China

E-mail: jl11269@buaa.edu.cn; xiaoxunli@buaa.edu.cn; k.li@camoptics.cn

Note 1. The cartoon illustration of random orientation and specific alignment of liquid crystal molecules in PDLC via voltage changing.


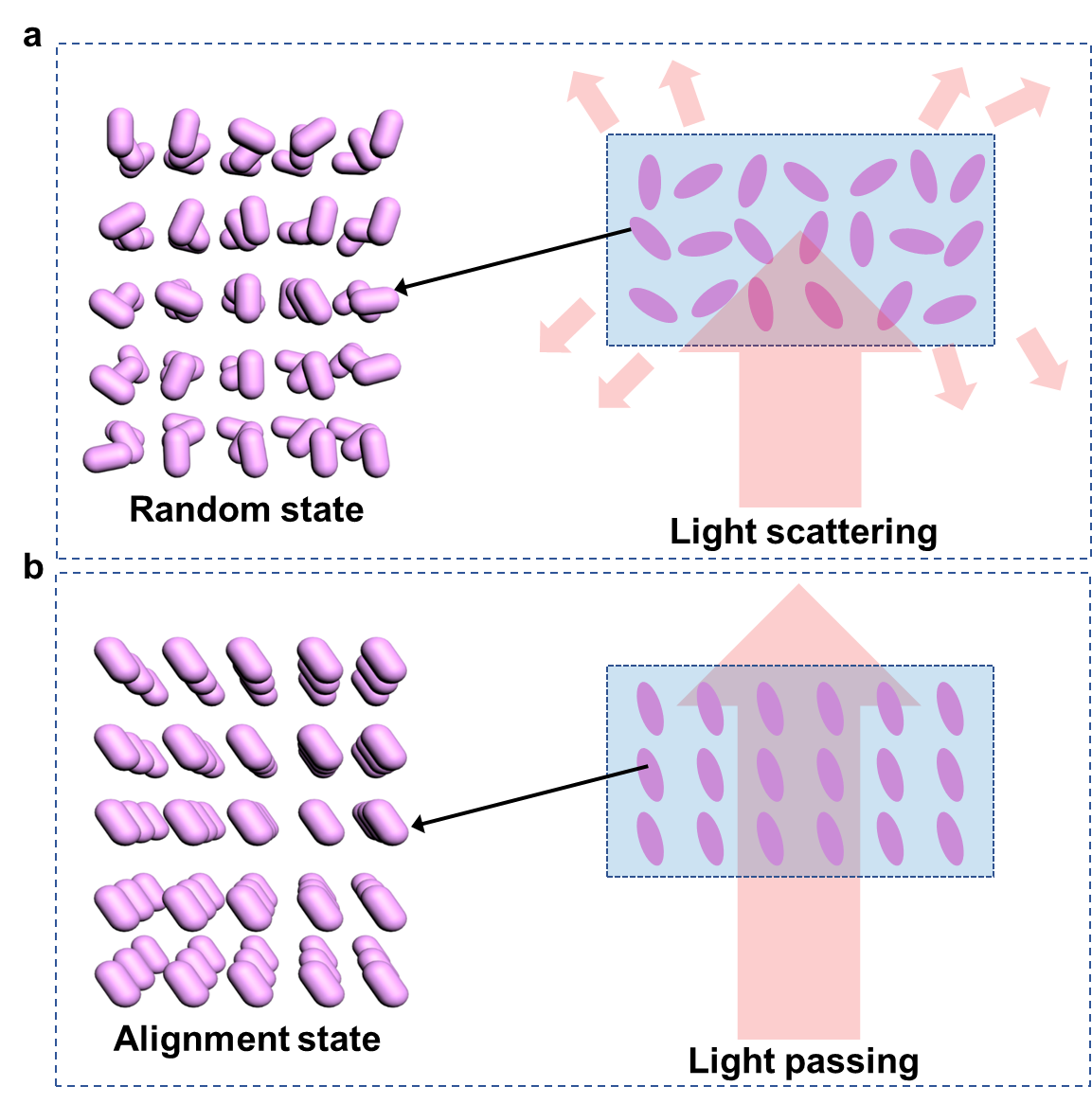
5

Fig. S1. The cartoon illustration of random orientation and specific alignment of liquid crystal molecules in PDLC via voltage changing. a Random state with light scattering property, b Alignment state with lighting passing property.

Note 2. Variations in light transmittance of PDLC with different thickness at 473 nm and 532 nm optical wavelength.


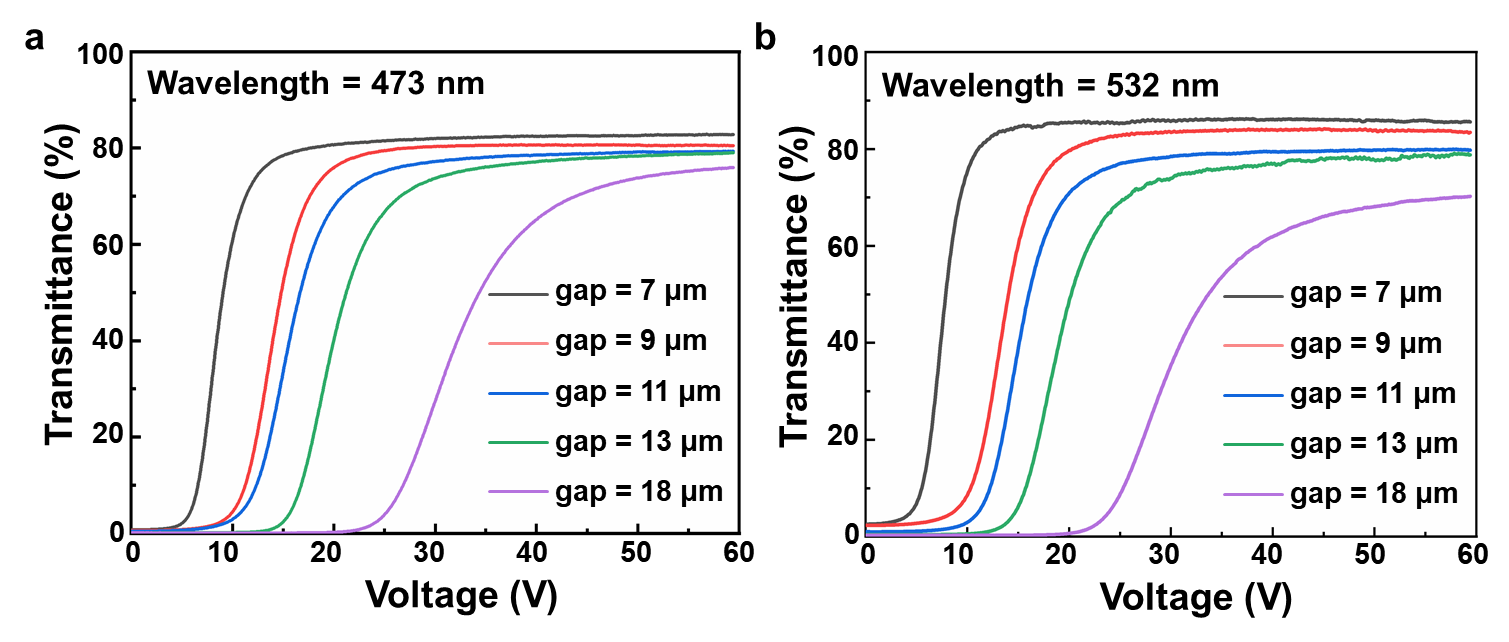


**Fig. S2.** The voltage-dependent transmittance curves of PDLC with different thickness at **a** 473 nm and **b** 532 nm optical wavelength.

Note 3. Drive voltage and saturation voltage of PDLC with different thickness at 473 nm and 532 nm optical wavelength.


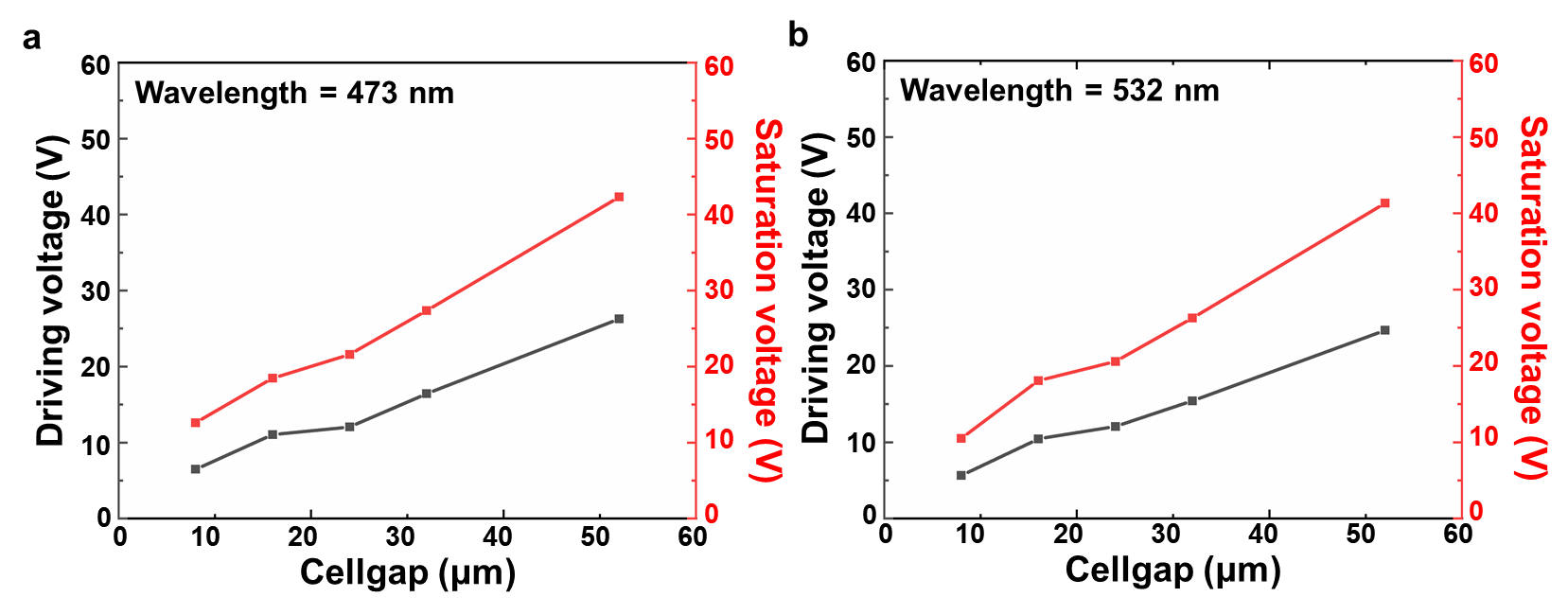


Fig. S3. The voltage-dependent transmittance curves of PDLC with different thickness at a 473 nm and b 532 nm optical wavelength.

Note 4. The response speed and repeated switching process of PDLC with different thickness at 473 nm and 532 nm optical wavelength.


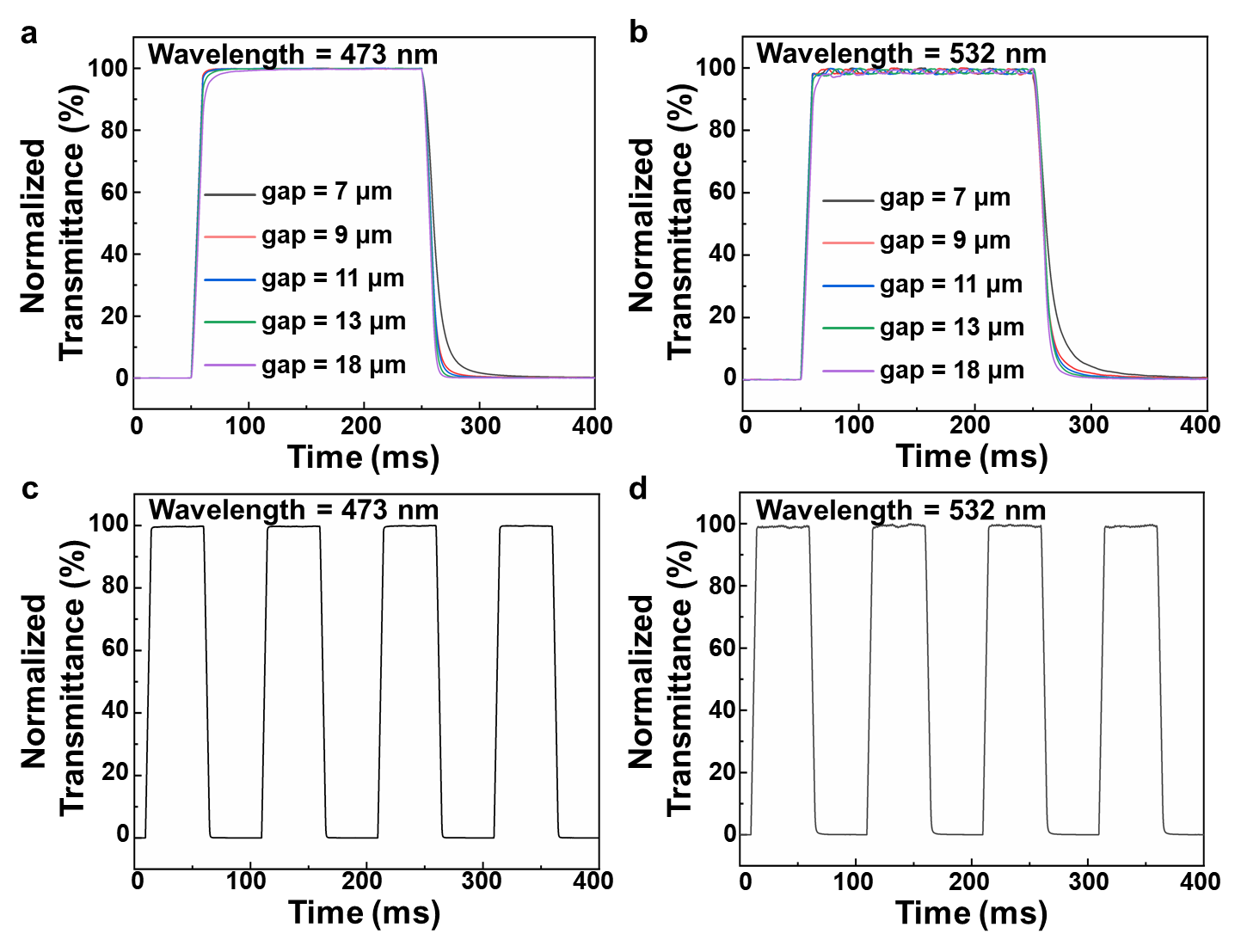


Fig. S4. The switching rate of PDLCs is characterized by the normalized transmittance at a 473 nm and b 532 nm optical wavelength. The repeated switching process of PDLC at c 473 nm and d 532 nm optical wavelength.

By quantifying the variations in transmittance response of PDLC with different thickness, we can ascertain the rise time, fall time, and response time at 473 nm (**Table S1**), 532 nm (**Table S2**) and 635 nm (**Table S3**) optical wavelength.

Table S1. Response speed of PDLC with different thickness at 473 nm optical wavelength.

| **Thickness/um** | **Rise Time/ms** | **Fall Time/ms** | **Response Time/ms** |
| --- | --- | --- | --- |
| 7 | 8.16 | 19.78 | 27.95 |
| 9 | 8.15 | 12.67 | 20.81 |
| 11 | 8.21 | 11.66 | 19.86 |
| 13 | 8.43 | 10.55 | 18.98 |
| 18 | 9.95 | 9.58 | 19.53 |

Table S2. Response speed of PDLC with different thickness at 532 nm optical wavelength.

| **Thickness/um** | **Rise Time/ms** | **Fall Time/ms** | **Response Time/ms** |
| --- | --- | --- | --- |
| 7 | 8.17 | 27.72 | 35.88 |
| 9 | 8.15 | 17.76 | 25.91 |
| 11 | 8.15 | 15.82 | 23.97 |
| 13 | 8.35 | 13.99 | 22.35 |
| 18 | 9.15 | 12.29 | 21.44 |

Table S3. Response speed of PDLC with different thickness at 633 nm optical wavelength.

| **Thickness/um** | **Rise Time/ms** | **Fall Time/ms** | **Response Time/ms** |
| --- | --- | --- | --- |
| 7 | 8.24 | 47.05 | 55.29 |
| 9 | 8.19 | 30.95 | 39.14 |
| 11 | 8.19 | 24.35 | 32.54 |
| 13 | 8.28 | 17.85 | 26.13 |
| 18 | 8.61 | 14.63 | 23.24 |

Note 5. The variations in transmittance of PDLC with different thickness under various wavelengths and voltages.


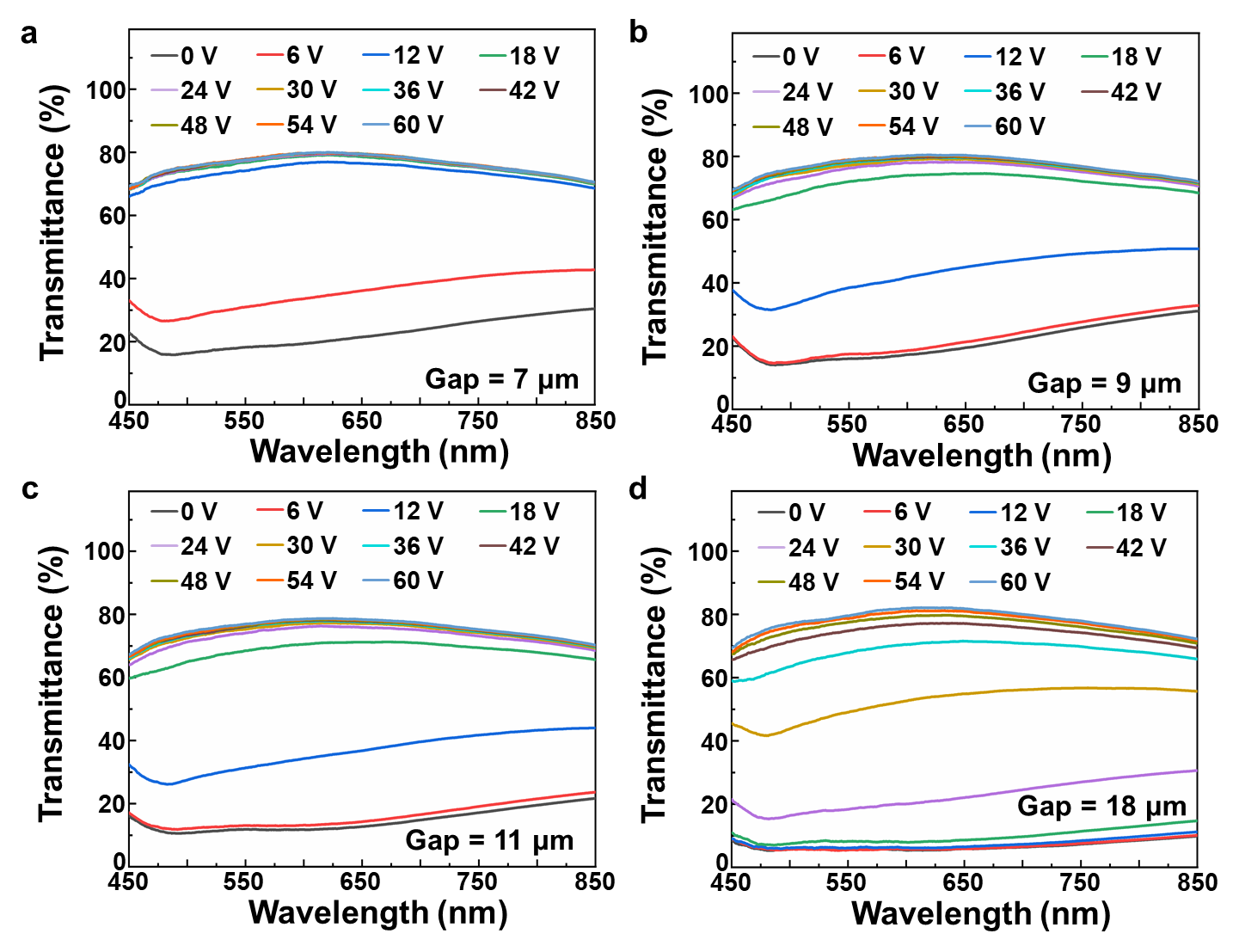


Fig. S5. The transmittance of PDLC exhibits continuous variations under different wavelengths, corresponding to distinct applied voltages. The thickness of PDLC is a 7 um, b 9 um, c 11 um, and d 18 um.

Note 6. The influence of PDLC on the polarization of incident light under different voltages.


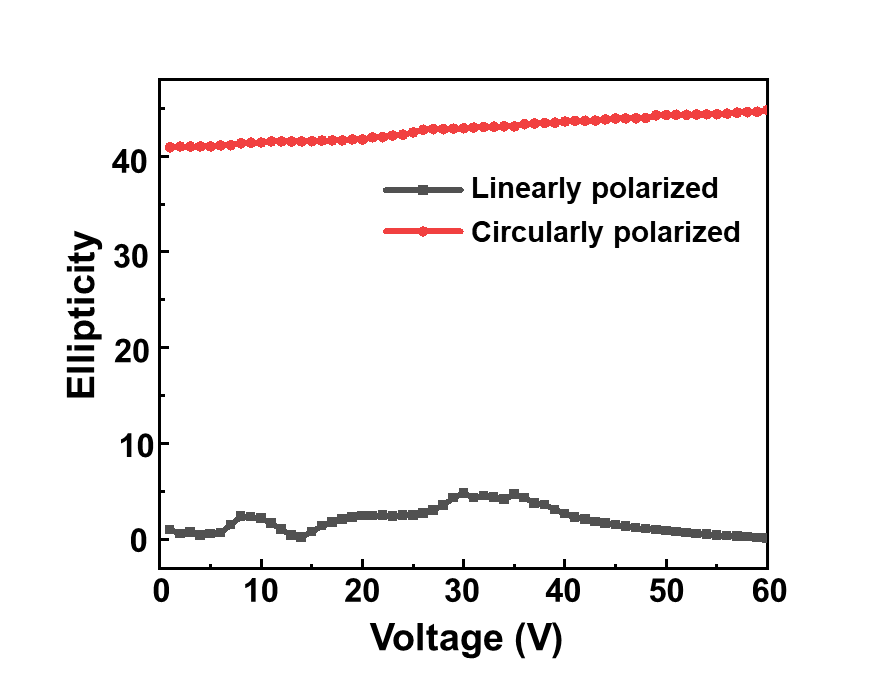


Fig. S6. Ellipticity of light after PDLC under different voltages by a Linearly polarized light incident and b Circularly polarized light incident.

Note 7. Variation in the transmittance of PDLC under different testing distances at 473 nm and 532 nm optical wavelength.


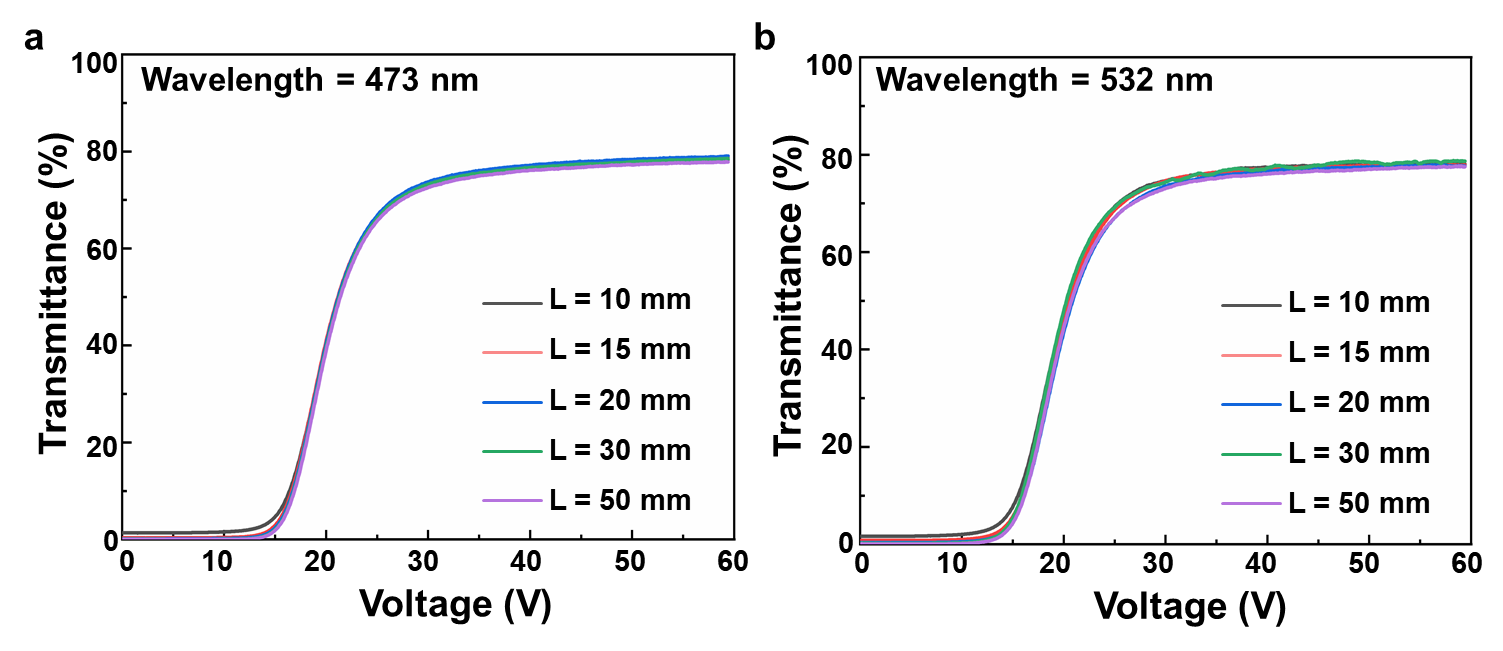


Fig. S7. The voltage-dependent transmittance of PDLC exhibits variations at different testing distances at a 473 nm and b 532 nm optical wavelength.

Note 8. The cross-polarization transmittance at 473 nm and 532 nm optical wavelength.


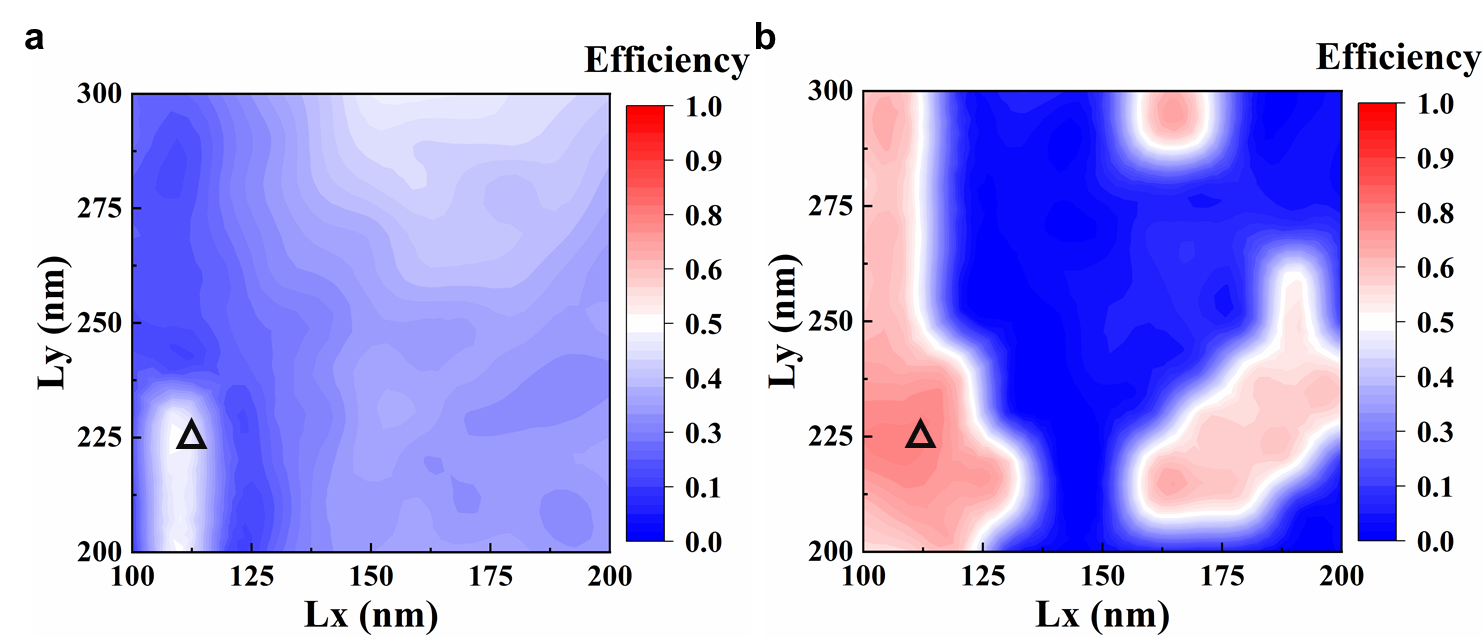


Fig. S8. Simulation graph of the cross-polarization transmittance of a rectangular nanopillar with different length-width ratio at a 473 nm and b 532 nm optical wavelength.

Note 9. The process of integrated PDLC-metasurface.


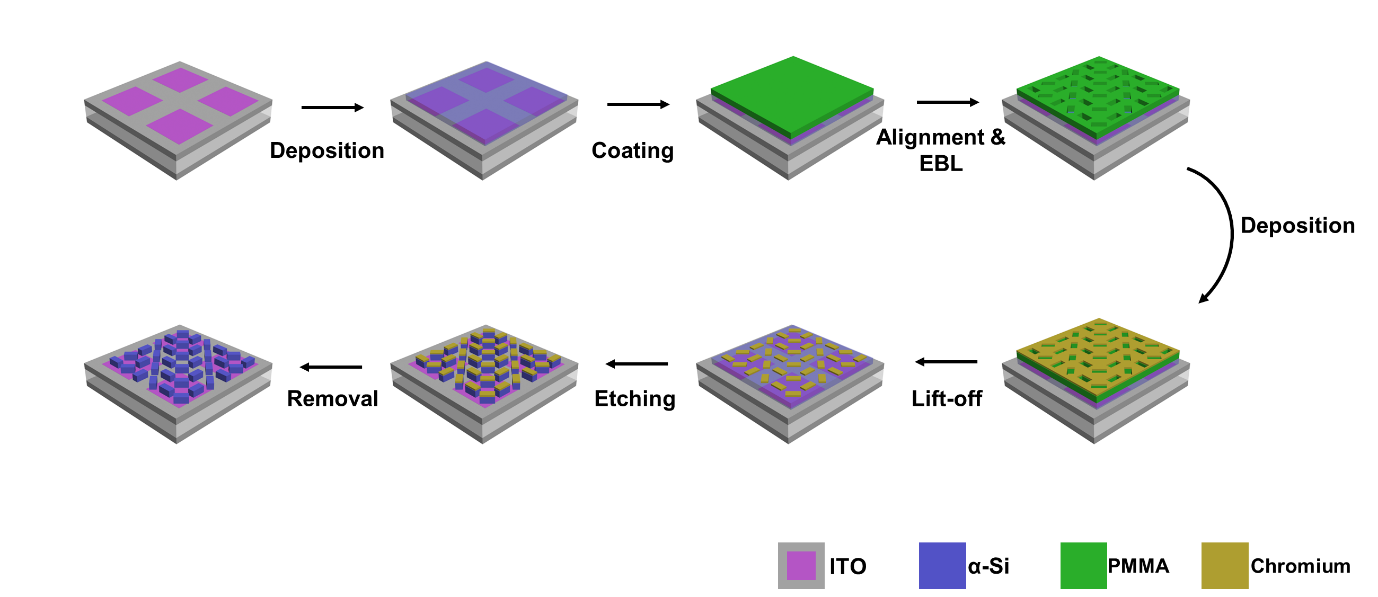


Fig. S9. The process diagram of integrated PDLC-metasurface. Metasurface was prepared on PDLC device.

Note 10. 3D metasurface holographic display at different optical wavelength.


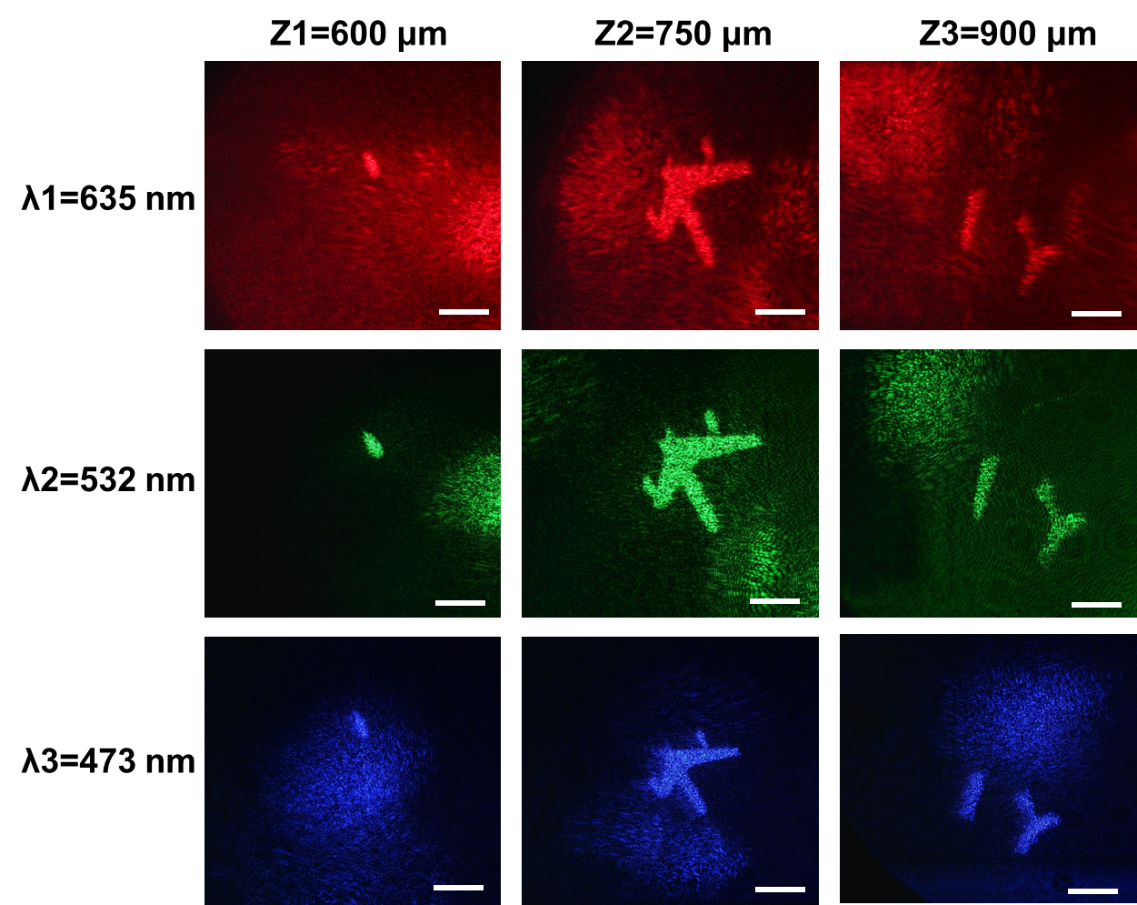


Fig. S10. 3D metasurface holographic display at different optical wavelength (λ) at different depths produced by integrating virtual holographic-lens of different focal lengths (Z) (the scale bar represents 0.5 cm).

Note 11. Efficiency measurement of metasurface holography.

The metasurface is illuminated with left-circularly polarized light, and the incident light power is measured using an optical power meter. The unit structure of the metasurface act as a half-wave plate (HWP) to generate holographic images with right-circularly polarization. Unconverted left-circularly polarized light is filtered out using quarter-wave plate (QWP) and linear polarizer (LP). The total optical power of the holographic image after zero-order filtering is determined using an optical power detector. The efficiency measurement of metasurface holography at different wavelengths is shown in Table S4.

Table S4. Efficiency measurement of metasurface holography at different optical wavelength.

| **Wavelength** | **Incident light power/mW** | | **Total optical power of holographic image/mW** | **Efficiency** |
| --- | --- | --- | --- | --- |
| 635 nm | | 40.12 | 26.75 | 66.68% |
| 532 nm | | 39.87 | 22.92 | 57.49% |
| 473 nm | | 40.45 | 19.61 | 48.48% |

Note 12. The holographic phase map and simulated reconstruction image of the metasurface.


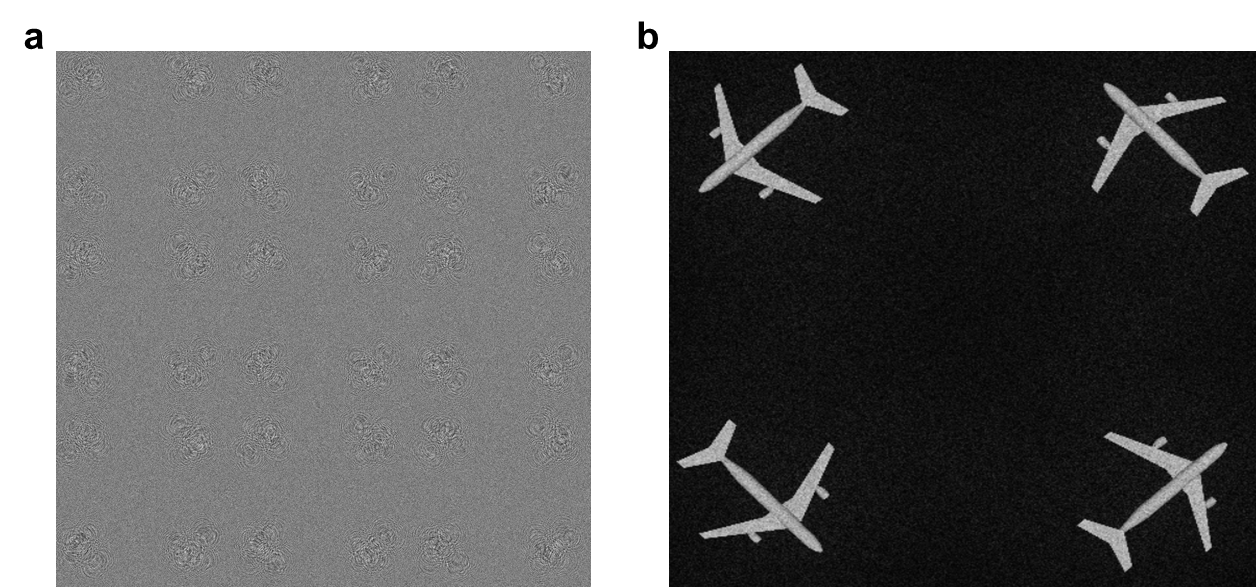


Fig. S11. a holographic phase map. b simulated reconstruction image.

Note 13. The PDLC-metasurface exhibiting holographic images and dynamic control responses at 473 nm and 532 nm optical wavelength.


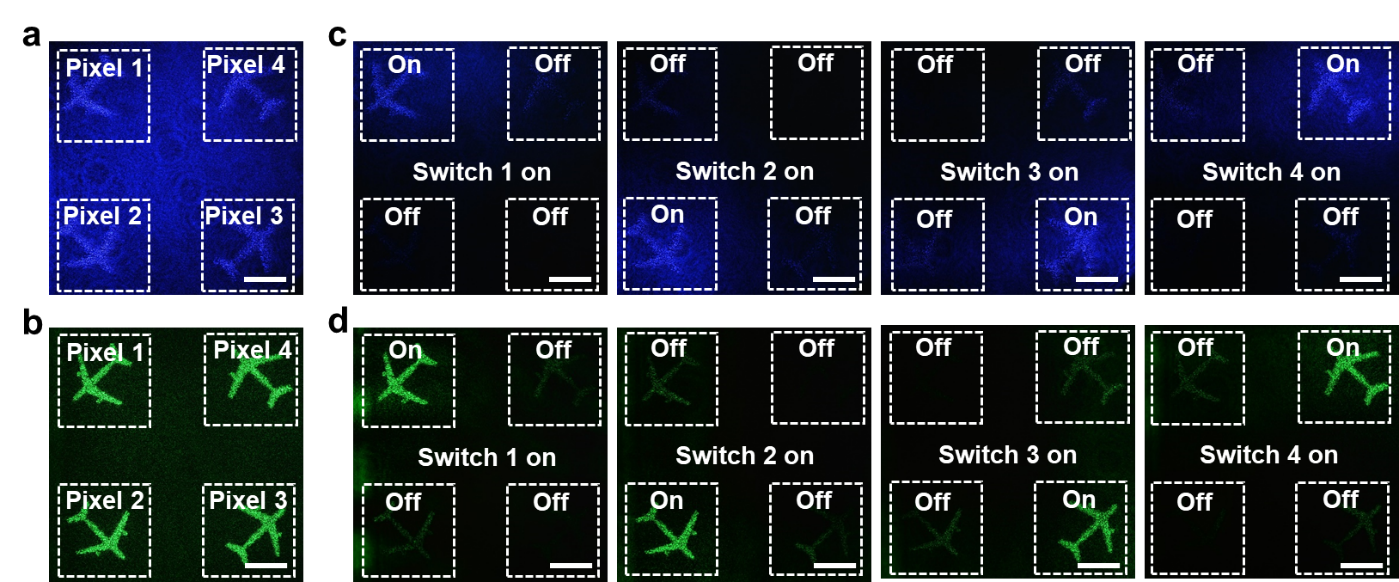


Fig. S12. A holographic display system with four channels at a 473 nm and b 532 nm optical wavelength (the scale bar represents 0.5 cm). Dynamic addressing sequentially controls the display of four-channel holographic images at c 473 nm and d 532 nm optical wavelength (the scale bar represents 0.5 cm).

Note 14. Multi-channel PDLC crosstalk measurement at 473 nm, 532 nm, and 635 nm optical wavelength.


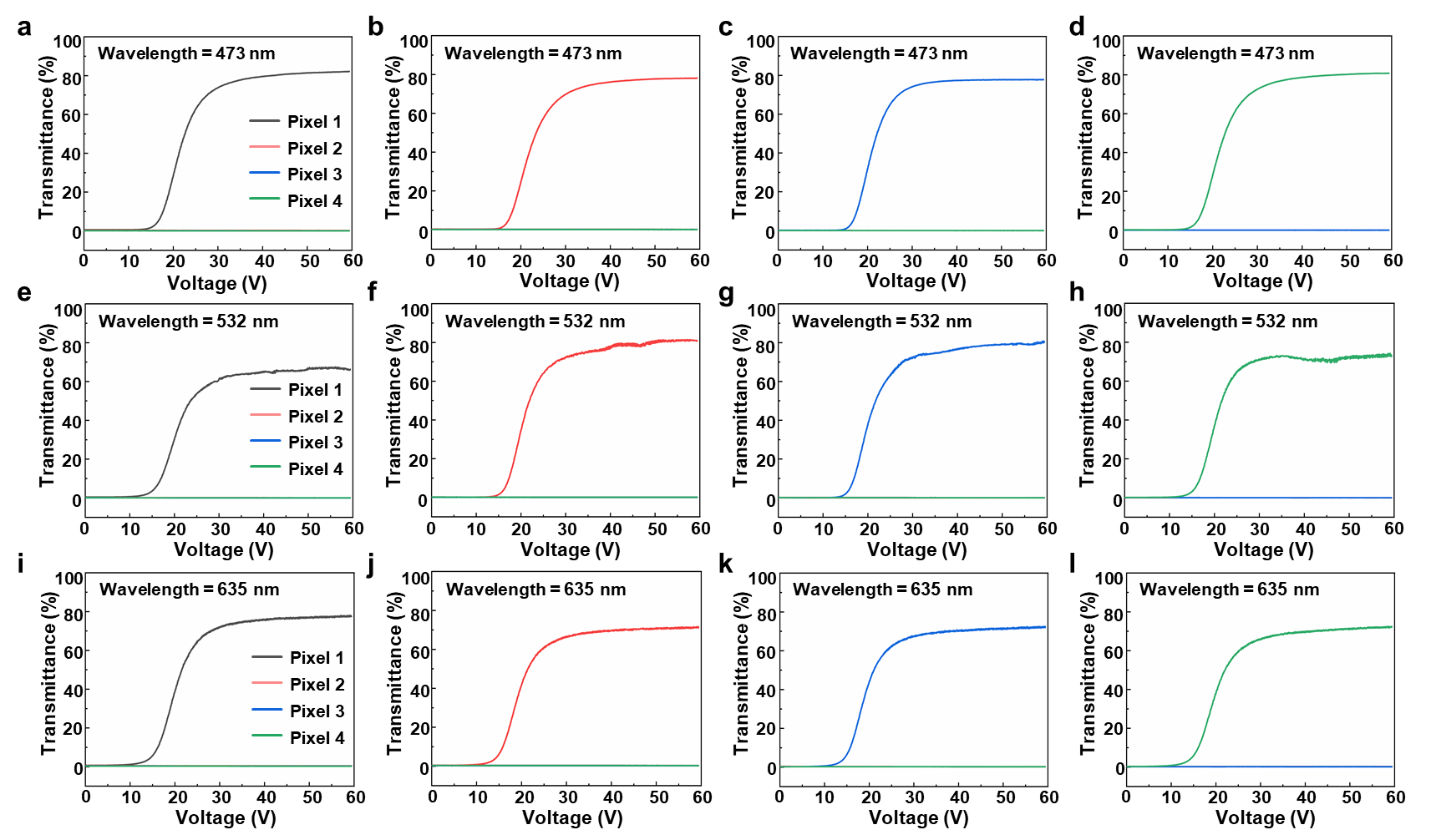


Fig. S13. Multi-channel PDLC crosstalk measurement at 473 nm, 532 nm and 635 nm optical wavelength. a The transmittance of each pixel at 473 nm varies with voltage when channel 1 is activated. b The transmittance of each pixel at 473 nm varies with voltage when channel 2 is activated. c The transmittance of each pixel at 473 nm varies with voltage when channel 3 is activated. d The transmittance of each pixel at 473 nm varies with voltage when channel 4 is activated. e The transmittance of each pixel at 532 nm varies with voltage when channel 1 is activated. f The transmittance of each pixel at 532 nm varies with voltage when channel 2 is activated. g The transmittance of each pixel at 532 nm varies with voltage when channel 3 is activated. h The transmittance of each pixel at 532 nm varies with voltage when channel 4 is activated. i The transmittance of each pixel at 635 nm varies with voltage when channel 1 is activated. j The transmittance of each pixel at 635 nm varies with voltage when channel 2 is activated. k The transmittance of each pixel at 635 nm varies with voltage when channel 3 is activated. l The transmittance of each pixel at 635 nm varies with voltage when channel 4 is activated.

The transmittance and crosstalk (described by the contrast between channels, defined as the ratio of the transmittance of an active channel to the transmittance of that channel) of each channel was measured when the four channels were activated one by one at 473 nm (Table S5), 532 nm (Table S6) and 635 nm (Table S7) optical wavelength.

Table S5. The transmittance and crosstalk of each channel at 473 nm optical wavelength.

| **Transmittance (contrast)** | **Channel 1** | **Channel 2** | **Channel 3** | **Channel 4** |
| --- | --- | --- | --- | --- |
| Channel 1 activated | 82.147% | 0.058%  (1422:1) | 0.020%  (4151:1) | 0.026%  (3218:1) |
| Channel 2 activated | 0.058%  (1356:1) | 78.177% | 0.050%  (1553:1) | 0.077%  (1011:1) |
| Channel 3 activated | 0.018%  (4277:1) | 0.038%  (2039:1) | 77.761% | 0.006%  (13407:1) |
| Channel 4 activated | 0.019%  (4325:1) | 0.025%  (3241:1) | 0.021%  (3856:1) | 80.786% |

Table S6. The transmittance and crosstalk of each channel at 532 nm optical wavelength.

| **Transmittance (contrast)** | **Channel 1** | **Channel 2** | **Channel 3** | **Channel 4** |
| --- | --- | --- | --- | --- |
| Channel 1 activated | 66.332% | 0.052%  (1277:1) | 0.042%  (1585:1) | 0.034%  (1931:1) |
| Channel 2 activated | 0.054%  (1498:1) | 80.855% | 0.051%  (1574:1) | 0.079%  (1022:1) |
| Channel 3 activated | 0.051%  (1591:1) | 0.028%  (2909:1) | 80.498% | 0.066%  (1228:1) |
| Channel 4 activated | 0.035%  (2054:1) | 0.004%  (17392:1) | 0.027%  (2662:1) | 72.698% |

Table S7. The transmittance and crosstalk of each channel at 532 nm optical wavelength.

| **Transmittance (contrast)** | **Channel 1** | **Channel 2** | **Channel 3** | **Channel 4** |
| --- | --- | --- | --- | --- |
| Channel 1 activated | 77.451% | 0.047%  (1640:1) | 0.033%  (2353:1) | 0.029%  (2674:1) |
| Channel 2 activated | 0.037%  (1942:1) | 71.525% | 0.022%  (3190:1) | 0.025%  (2848:1) |
| Channel 3 activated | 0.024%  (2974:1) | 0.028%  (2597:1) | 72.416% | 0.025%  (2861:1) |
| Channel 4 activated | 0.026%  (2740:1) | 0.072%  (1009:1) | 0.069%  (1045:1) | 72.337% |

Note 15. Multi-channel integrated PDLC-metasurface holographic display crosstalk measurement at 473 nm, 532 nm, and 635 nm optical wavelength.

The total optical power of zero-order filtered metasurface holographic image in activated channels and deactivated channels is measured at 473 nm (Table S8), 532 nm (Table S9), and 635 nm (Table S10) optical wavelength, and the crosstalk (described by the contrast between channels, defined as the ratio of the total optical power of the zero-order filtered metasurface holographic image to the optical power of that channel) between channels is analyzed.

Table S8. The optical power and crosstalk of holographic image and other channels at 473 nm optical wavelength.

| **Optical power/mW (contrast)** | **Channel 1** | **Channel 2** | **Channel 3** | **Channel 4** |
| --- | --- | --- | --- | --- |
| Channel 1 activated | 4.029 | 0.104  (39:1) | 0.094  (43:1) | 0.129  (31:1) |
| Channel 2 activated | 0.115  (34:1) | 3.892 | 0.116  (33:1) | 0.087  (45:1) |
| Channel 3 activated | 0.082  (47:1) | 0.120  (32:1) | 3.885 | 0.131  (30:1) |
| Channel 4 activated | 0.158  (26:1) | 0.098  (42:1) | 0.104  (40:1) | 4.115 |

Table S9. The optical power and crosstalk of holographic image and other channels at 532 nm optical wavelength.

| **Optical power/mW (contrast)** | **Channel 1** | **Channel 2** | **Channel 3** | **Channel 4** |
| --- | --- | --- | --- | --- |
| Channel 1 activated | 3.871 | 0.137  (28:1) | 0.094  (41:1) | 0.159  (24:1) |
| Channel 2 activated | 0.189  (25:1) | 4.812 | 0.173  (28:1) | 0.123  (39:1) |
| Channel 3 activated | 0.119  (40:1) | 0.171  (28:1) | 4.808 | 0.221  (22:1) |
| Channel 4 activated | 0.159  (28:1) | 0.094  (47:1) | 0.123  (36:1) | 4.440 |

Table S10. The optical power and crosstalk of holographic image and other channels at 635 nm optical wavelength.

| **Optical power/mW (contrast)** | **Channel 1** | **Channel 2** | **Channel 3** | **Channel 4** |
| --- | --- | --- | --- | --- |
| Channel 1 activated | 5.620 | 0.191  (29:1) | 0.126  (45:1) | 0.156  (36:1) |
| Channel 2 activated | 0.186  (27:1) | 5.037 | 0.159  (32:1) | 0.127  (40:1) |
| Channel 3 activated | 0.133  (38:1) | 0.188  (27:1) | 5.039 | 0.171  (29:1) |
| Channel 4 activated | 0.157  (33:1) | 0.139  (37:1) | 0.149  (34:1) | 5.122 |

In addition, based on the holographic images (Fig. 6c-f and Fig. S12c, d), the crosstalk between channels can also be analyzed. The holographic images were divided by channel and the average pixel intensity (defined as the ratio of the total intensity of the holographic image to the number of pixels) was calculated by Matlab. At 473 nm (Table S11), 532 nm (Table S12), and 635 nm (Table S13) optical wavelength, the average pixel intensity and crosstalk (described by the contrast between channels, defined as the ratio of the average pixel intensity of holographic image to the average pixel intensity of that channel) of holographic images in different channels are shown. The reason why the contrast of this calculation method is lower than that of the optical power method may be due to the exposure of the camera when capturing holographic images.

Table S11. The average pixel intensity and crosstalk of holographic image and other channels at 473 nm optical wavelength.

| **Average pixel intensity (contrast)** | **Channel 1** | **Channel 2** | **Channel 3** | **Channel 4** |
| --- | --- | --- | --- | --- |
| Channel 1 activated | 25.084 | 1.315  (19:1) | 1.165  (22:1) | 1.462  (17:1) |
| Channel 2 activated | 1.352  (18:1) | 24.852 | 1.404  (18:1) | 1.175  (21:1) |
| Channel 3 activated | 1.127  (22:1) | 1.742  (14:1) | 24.689 | 1.947  (13:1) |
| Channel 4 activated | 1.816  (14:1) | 1.017  (26:1) | 1.160  (22:1) | 25.985 |

Table S12. The average pixel intensity and crosstalk of holographic image and other channels at 532 nm optical wavelength.

| **Average pixel intensity (contrast)** | **Channel 1** | **Channel 2** | **Channel 3** | **Channel 4** |
| --- | --- | --- | --- | --- |
| Channel 1 activated | 26.798 | 1.842  (15:1) | 1.248  (21:1) | 1.896  (14:1) |
| Channel 2 activated | 2.156  (13:1) | 27.249 | 1.918  (14:1) | 1.246  (22:1) |
| Channel 3 activated | 1.160  (24:1) | 1.520  (18:1) | 27.313 | 1.720  (16:1) |
| Channel 4 activated | 1.561  (17:1) | 1.017  (27:1) | 1.315  (21:1) | 26.994 |

Table S13. The average pixel intensity and crosstalk of holographic image and other channels at 635 nm optical wavelength.

| **Average pixel intensity (contrast)** | **Channel 1** | **Channel 2** | **Channel 3** | **Channel 4** |
| --- | --- | --- | --- | --- |
| Channel 1 activated | 27.197 | 1.819  (15:1) | 1.113  (24:1) | 1.592  (17:1) |
| Channel 2 activated | 1.719  (15:1) | 26.012 | 1.542  (17:1) | 1.295  (20:1) |
| Channel 3 activated | 1.192  (22:1) | 1.405  (19:1) | 26.113 | 1.320  (20:1) |
| Channel 4 activated | 1.320  (20:1) | 1.110  (24:1) | 1.322  (20:1) | 26.229 |

Note 16. The periodic switching modulation of each pixel of PDLC-metasurface at 473 nm and 532 nm optical wavelength.


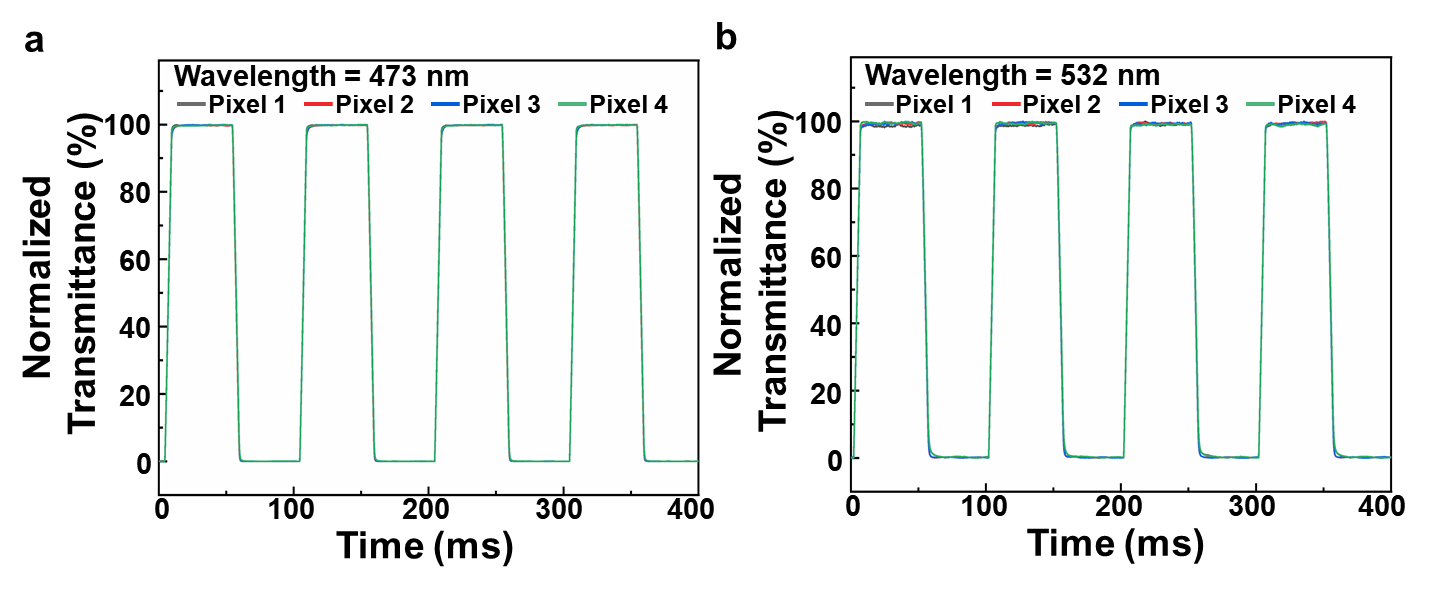


Fig. S14. The transmittance response through the four-pixel channel is cyclically modulated, resulting in periodic switching on and off states at a 473 nm and b 532 nm optical wavelength.

By quantifying the variations in transmittance response of each pixel of PDLC-metasurface, we can ascertain the rise time, fall time, and response time at 473 nm (**Table S14**), 532 nm (**Table S15**) and 635 nm (**Table S16**) optical wavelength.

Table S14. Response speed of each pixel of PDLC-metasurface at 473 nm optical wavelength.

| **Pixel** | **Rise Time/ms** | **Fall Time/ms** | **Response Time/ms** |
| --- | --- | --- | --- |
| 1 | 8.445 | 11.525 | 19.970 |
| 2 | 10.435 | 10.955 | 21.390 |
| 3 | 9.920 | 11.470 | 21.390 |
| 4 | 8.495 | 12.230 | 20.725 |

Table S15. Response speed of each pixel of PDLC-metasurface at 532 nm optical wavelength.

| **Pixel** | **Rise Time/ms** | **Fall Time/ms** | **Response Time/ms** |
| --- | --- | --- | --- |
| 1 | 8.285 | 17.060 | 25.345 |
| 2 | 8.920 | 14.445 | 23.365 |
| 3 | 8.875 | 14.940 | 23.815 |
| 4 | 8.325 | 17.905 | 26.230 |

Table S16. Response speed of each pixel of PDLC-metasurface at 633 nm optical wavelength.

| **Pixel** | **Rise Time/ms** | **Fall Time/ms** | **Response Time/ms** |
| --- | --- | --- | --- |
| 1 | 8.280 | 17.930 | 26.210 |
| 2 | 8.545 | 20.970 | 29.515 |
| 3 | 8.595 | 20.850 | 29.445 |
| 4 | 8.390 | 18.515 | 26.905 |

Note 17. Comparison of this work with other electrically controlled dynamic metasurface holography.

Compare the response time, contrast, and continuous adjustment of this work with other electrically controlled dynamic metasurface holography, as shown in Table S17 (Performance not explicitly specified is represented by “-”.).

Table S17. Comparison of this work with other electrically controlled dynamic metasurface holography.

| **Wavelength** | **2D/3D** | **Response time** | **Contrast** | **Continuous adjustment** | **Reference** |
| --- | --- | --- | --- | --- | --- |
| 473 nm, 532 nm, 635 nm | 3D | 19.97 ms | ＞1009:1 (device), ＞13:1 (total holographic image) | Yes | Our work |
| 635 nm | 2D | 105 ms | 105:1 (device) | Yes | 1 |
| 520 nm, 633 nm | 2D | 83 ms | 860:1 (device), ca. 220 (a line of holographic image) | No | 2 |
| 458 nm, 532 nm, 600 nm, 633 nm | 2D | 20-30 ms | - | No | 3 |
| 550 nm, 600 nm, 650 nm, 680 nm | 2D | 27 ms | - | No | 4 |
| 520 nm, 633 nm | 2D | 100 ms | - | No | 5 |
| 633 nm | 2D | >100 s | - | No | 6 |
| 633 nm | 2D | 350 s | - | No | 7 |
| 470 nm, 540 nm, 633 nm | 2D | seconds level | - | Yes | 8 |
| 420 nm, 450 nm 480 nm, 510 nm, 540 nm, 570 nm, 600 nm, 640 nm, 680 nm, 720 nm | 2D | - | - | Yes | 9 |
| 532 nm | 2D | - | - | No | 10 |
| 325 nm, 405 nm, 473 nm, 532 nm | 2D | - | - | No | 11 |
| 635 nm | 2D | - | - | Yes | 12 |
| 633 nm | 2D | - | - | No | 13 |

**References**

1. Li, J., Yu, P., Zhang, S. & Liu, N. Electrically-controlled digital metasurface device for light projection displays. *Nat. Commun.* **11**, 3574 (2020).
2. Kaissner, R. et al. Electrochemically controlled metasurfaces with high-contrast switching at visible frequencies. *Sci. Adv.* **7**, eabd9450 (2021).
3. Kim, I. et al. Stimuli‐responsive dynamic metaholographic displays with designer liquid crystal modulators. *Adv. Mater.* **32**, 2004664 (2020).
4. Wan, C. et al. Electric-driven meta-optic dynamics for simultaneous near-/far-field multiplexing display. *Adv. Funct. Mater.* **32**, 2110592 (2022).
5. Yu, P., Li, J. & Liu, N. Electrically tunable optical metasurfaces for dynamic polarization conversion. *Nano Lett.* **21**, 6690-6695 (2021).
6. Li, J. et al. Addressable metasurfaces for dynamic holography and optical information encryption. *Sci. Adv.* **4**, eaar6768 (2018).
7. Li, J., Chen, Y., Hu, Y., Duan, H. & Liu, N. Magnesium-based metasurfaces for dual-function switching between dynamic holography and dynamic color display. *ACS Nano* **14**, 7892-7898 (2020).
8. Wang, Z. et al. Vectorial liquid-crystal holography. *eLight* **4**, 5 (2024).
9. Kim, J. et al. Dynamic hyperspectral holography enabled by inverse-designed metasurfaces with oblique helicoidal cholesterics. *Adv. Mater.* **36**, 2311785 (2024).
10. Hu, Y. et al. Electrically tunable multifunctional polarization-dependent metasurfaces integrated with liquid crystals in the visible region. *Nano Lett.* **21**, 4554-4562 (2021).
11. Asad, A. et al. Spin-isolated ultraviolet-visible dynamic meta-holographic displays with liquid crystal modulators. *Nanoscale Horiz.* **8**, 759-766 (2023).
12. Yang, Y., Kim, H., Badloe, T. & Rho, J. Gap-plasmon-driven spin angular momentum selection of chiral metasurfaces for intensity-tunable metaholography working at visible frequencies. *Nanophotonics* **11**, 4123-4133 (2022).
13. Wang, J. et al. Cholesteric liquid crystal-enabled electrically programmable metasurfaces for simultaneous near-and far-field displays. *Nanoscale* **14**, 17921-17928 (2022).
